# Identification of a DUF538 gene conferring resistance to tea green leafhopper (*Empoasca onukii*) in *Camellia sinensis*

**DOI:** 10.1093/hr/uhaf264

**Published:** 2025-10-01

**Authors:** Yinghao Wang, Chunlei Ma, Xiangrui Kong, Yaodong Zheng, Feiruoran Aikeremu, Minsheng You, Liang Chen, Qian Zhao

**Affiliations:** State Key Laboratory of Agricultural and Forestry Biosecurity, Institute for Applied Ecology, Fujian Agriculture and Forestry University, Fuzhou 350002, China; National Key Laboratory for Tea Plant Germplasm Innovation and Resource Utilization, Tea Research Institute of the Chinese Academy of Agricultural Sciences, Hangzhou 310008, China; Institute of Tea, Fujian Academy of Agricultural Sciences, Fuzhou 350000, China; State Key Laboratory of Agricultural and Forestry Biosecurity, Institute for Applied Ecology, Fujian Agriculture and Forestry University, Fuzhou 350002, China; State Key Laboratory of Agricultural and Forestry Biosecurity, Institute for Applied Ecology, Fujian Agriculture and Forestry University, Fuzhou 350002, China; State Key Laboratory of Agricultural and Forestry Biosecurity, Institute for Applied Ecology, Fujian Agriculture and Forestry University, Fuzhou 350002, China; International Joint Research Laboratory of Ecological Pest Control, Ministry of Education, Fujian Agriculture and Forestry University, Fuzhou 350002, China; National Key Laboratory for Tea Plant Germplasm Innovation and Resource Utilization, Tea Research Institute of the Chinese Academy of Agricultural Sciences, Hangzhou 310008, China; State Key Laboratory of Agricultural and Forestry Biosecurity, Institute for Applied Ecology, Fujian Agriculture and Forestry University, Fuzhou 350002, China; International Joint Research Laboratory of Ecological Pest Control, Ministry of Education, Fujian Agriculture and Forestry University, Fuzhou 350002, China

Dear Editor,

Tea cultivation history spanning nearly 2000 years, stands as one of the world’s oldest crops and the most widely consumed non-alcoholic beverage globally. The tea plants play a pivotal role in rural development, alleviating poverty, and sustaining the livelihoods of millions of small-scale farmers. Global tea production exceeds 6.7 million tons with an economic value over US$17 billion (FAO: https://www.fao.org/). As the tea industry has developed, pest infestations have increased, with ~84% of agricultural chemicals applied in tea gardens being insecticides. Among tea pests, *Empoasca onukii* Matsuda stands out as the most prevalent and challenging to manage, causing yield losses of 15% to 50% [1]. Approximately 56% of insecticides used in tea plantations target this particular insect, resulting in quality and safety issues, such as excessive pesticide residues [2]. Female leafhoppers lay eggs in hidden render stems, making chemical pesticides less effective. Therefore, identifying quantitative trait loci and genes conferring resistance to *E. onukii* will serve as the foundation for resistance breeding in *C. sinensis.*

**Figure 1:**
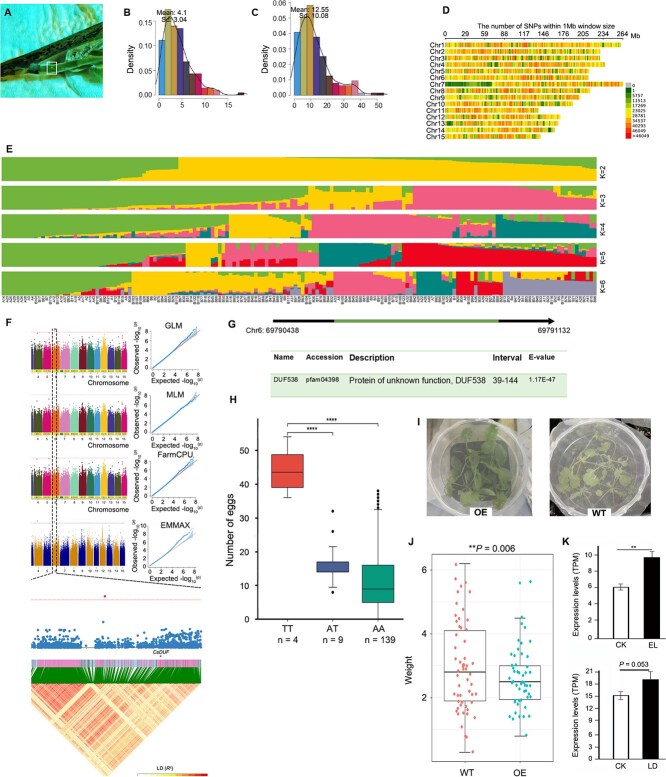
Role of *CsDUF* in modulating tea plant susceptibility to *E. onukii* infestation. (A) Detected eggs are marked with square box. (B) Mean egg counts per sample (± SE). (C) Maximum egg counts per sample (± SE). (D) Density distribution of SNPs across the genome, calculated in 1 Mb sliding windows. (E) Population structure separating the accessions into three subgroups (*K* = 2–6, with an optimal *K* = 3). (F) Manhattan plots of SNPs on 15 tea chromosomes. The *X*-axis indicates the genomic coordinates, and the *Y*-axis indicates the association score of each SNP; the score represents a transformed *P* values, −log10P. QQ plots for the egg-laying prevention of tea plants were also provided based on GLM, MLM, FarmCPU, and EMMAX models. (G) Gene structure of *CsDUF* gene in *Camellia sinensis.* (H) Boxplot showing the three genotypes at the target locus on chromosome 6. Statistical significance was assessed using Student’s *t*-test, with ^****^ indicating *P* < 0.0001. (I) *Arabidopsis thaliana* transformation and subsequent herbivory infestation. (J) Comparison of larval weight raised on control (WT) and transgenic (OE) plants. Statistical significance was assessed using Student’s *t*-test, with ^**^ indicating *P* < 0.01. A total of 60 larvae were used in this analysis. (K) *CsDUF* expression levels in tea plants following infestation by *E. oblique* and *E. onukii*. CK: control leaves (no herbivory); EL: leaves infested by *E. oblique* until 1/3 of each leaf was consumed; LD: plants exposed to ~100 *E. onukii* individuals, replenished every two days for 3 weeks (leafhopper-damaged tea samples). Statistical significance was assessed using Student's *t*-test, with ^**^ indicating *P* < 0.01*.*

We conducted a systematic survey to tally the number of eggs laid by *E. onukii* on 165 tea accessions that represent a significant proportion of superior *C. sinensis* cultivars from the National Tea Germplasm Repository. For each cultivar, 10 fresh shoots with one-bud-four-leaves were collected using a five-point sampling method over 15 days, with a total of 150 shoots sampled per cultivar. Eggs were detected using a blue-light source (460 nm wavelength) and blue-light filtering glasses (Fig. 1A). Our phenotypic evaluation revealed significant variation in *E. onukii* oviposition behavior across the tea cultivars. The highest number of eggs was observed on the Fuan Dabaicha cultivar (average, 12.91 eggs per 10 shoots; maximum, 54 eggs), while the fewest eggs were recorded on JP07 and Echa2 cultivars (average of 0.1 and 0 eggs, respectively). The phenotypic values follow a normal distribution (Fig. 1B and C), demonstrating significant variation in oviposition behavior across the tea cultivar populations.

Genomic DNA was extracted from young leaves using the CTAB method, and libraries with 350-bp inserts were sequenced on the DNBSEQ-T7 platform at 20× depth. Genome sequencing generated an average depth per sample of 21.77×, identifying 79 027 708 single nucleotide polymorphisms (SNPs), 2 740 936 insertions, and 3 910 027 deletions (Fig. 1D). Population structure was assessed using ADMIXTURE software (Fig. 1E), and GWAS was performed using general linear model, mixed linear model, FarmCPU, and EMMAX methods with a suggestive threshold *P*-value less than 3.5E−10.

The results of our genome-wide association study and linkage disequilibrium analysis revealed one significant SNP on chromosome 6 associated with preventing egg-laying in *E. onukii* (Fig. 1F)*.* Within a 50 kb region surrounding this trait-related SNP locus, we identified a *DUF538* gene (Domain of Unknown Function 538), tentatively designated as *CsDUF* (Fig. 1G). The favorable allelic variation at this SNP locus was the ‘TT’ genotype, which was associated with significantly reduced egg-laying by *E. onukii* (Fig. 1H). The DUF538 protein family is a conserved yet functionally enigmatic group implicated in plant stress responses, potentially through phosphorylation-mediated signaling modulation [3]. These proteins enhance the activity of redox enzymes such as catalase, peroxidase, polyphenol oxidase, and phenylalanine ammonia lyase, which may influence pathogen resistance via mechanisms analogous to bactericidal/permeability-increasing (BPI) proteins [3]. The observed larval weight reduction following *CsDUF* overexpression could stem from its role in boosting ROS-scavenging enzymes, consistent with catalase’s function in maintaining ROS homeostasis—its suppression leads to hydrogen peroxide accumulation and hypersensitive response activation. The stress-responsive nature of DUF538 proteins is further supported by their differential expression in various plant-pest systems, including tea plants infested by *E. onukii* [4]. Collectively, our findings suggest that *CsDUF* contributes to plant defense pathways, positioning DUF proteins as regulators in biotic stress responses.

To validate the function of this gene, we cloned the full-length CDS sequence of the *CsDUF* gene into an overexpression vector and transformed *Arabidopsis thaliana* using the floral dip method. First-instar larvae of diamondback moth were transferred to the transgenic plants, with each plant hosting five larvae (Fig. 1I). After 10 days, larvae feeding on plants overexpressing the *CsDUF* gene (OE) showed significantly reduced weight compared to those feeding on control plants (WT) (Fig. 1J). This functional validation confirms that the *CsDUF* gene contributes to insect resistance, likely through mechanisms that affect insect development and survival. RNA-seq analysis of leaves infested by tea pests revealed that the *CsDUF* gene was differentially expressed following pest attack (Fig. 1K), further supporting its role in the plant’s defense response.

GWAS is a powerful tool for dissecting the genetic basis of crop traits and advancing precision agriculture. In *Eucalyptus*, GWAS revealed 35 loci conferring resistance to *Leptocybe invasa* [5]. These findings confirmed GWAS as an effective method for mapping insect resistance genes in plants. Here, our GWAS analysis suggests that the *CsDUF* gene may contribute to resistance against *E. onukii*, potentially offering new insights for tea breeding research. This discovery provides valuable genetic material for understanding the molecular mechanisms of pest resistance in tea plants and offers a promising target for developing durably resistant tea cultivars. The favorable ‘TT’ genotype at the identified SNP locus can serve as a molecular marker for selecting resistant germplasm in breeding programs, potentially accelerating the development of new cultivars with enhanced resistance to this devastating pest.

Our findings have important implications for sustainable tea production. By incorporating the *CsDUF* gene into breeding programs, it may be possible to reduce reliance on chemical pesticides, thereby improving the quality and safety of tea products while reducing production costs and environmental impact. The DUF538 protein family is found across various plant species, but its precise function remains poorly understood [3]. Our study provides evidence that this protein family plays a role in plant defense against insect pests. The reduced weight of pest larvae feeding on plants overexpressing *CsDUF* suggests that this gene may affect insect development through antibiosis mechanisms, possibly by producing compounds that are toxic or anti-nutritional to the pest.

In conclusion, our genome-wide association study has successfully identified a genetic locus and candidate gene associated with resistance to *E. onukii* in tea plants. The *CsDUF* gene represents a promising target for marker-assisted selection in tea breeding programs aimed at developing resistant cultivars. Further research into the molecular function of this gene will enhance our understanding of plant-insect interactions and contribute to the development of sustainable pest management strategies for the tea industry worldwide.
